# Remote cortical perturbation dynamically changes the network solutions to given tactile inputs in neocortical neurons

**DOI:** 10.1016/j.isci.2021.103557

**Published:** 2021-12-02

**Authors:** Leila Etemadi, Jonas M.D. Enander, Henrik Jörntell

**Affiliations:** 1 Neural Basis of Sensorimotor Control, Department of Experimental Medical Science, Lund University, BMC F10 Tornavägen 10, 221 84 Lund, Sweden

**Keywords:** Neuroscience, Cellular neuroscience, Sensory neuroscience

## Abstract

The neocortex has a globally encompassing network structure, which for each given input constrains the possible combinations of neuronal activations across it. Hence, its network contains solutions. But in addition, the cortex has an ever-changing multidimensional internal state, causing each given input to result in a wide range of specific neuronal activations. Here we use intracellular recordings in somatosensory cortex (SI) neurons of anesthetized rats to show that remote, subthreshold intracortical electrical perturbation can impact such constraints on the responses to a set of spatiotemporal tactile input patterns. Whereas each given input pattern normally induces a wide set of preferred response states, when combined with cortical perturbation response states that did not otherwise occur were induced and consequently made other response states less likely. The findings indicate that the physiological network structure can dynamically change as the state of any given cortical region changes, thereby enabling a rich, multifactorial, perceptual capability.

## Highlights

• Tactile sensory input patterns evoke multi-structure cortical neuron responses
• Multi-structure responses are shown to be impacted by remote cortical regions
• Highly dynamic neuron responses reflects global cortical information integration
• Perception hence depends on globally distributed activity at the time of input

## Graphical abstract

**Figure:**
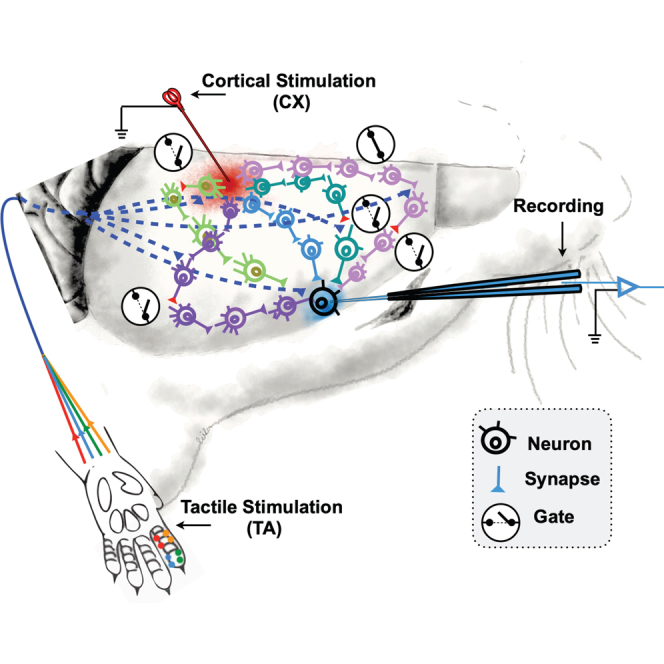

## Introduction

Several studies have reported a wide distribution of cortical activity in response to input from a variety of modalities, indicating that the neocortex is in principle a globally interconnected network (Ferezou et al., 2007; Frostig et al., 2008; Fu et al., 2003; Ghazanfar and Schroeder, 2006; Hihara et al., 2015; Keller et al., 2012; Olcese et al., 2013; Rancz et al., 2015; Saleem et al., 2013). More surprisingly, information about the specific quality of tactile inputs is present in apparently any region of the neocortex (Enander and Jorntell, 2019; Enander et al., 2019; Genna et al., 2018), and thalamus (Wahlbom et al., 2021), and remote stroke-like lesions degrade the processing of tactile inputs in neurons in the primary somatosensory cortex (SI) (Wahlbom et al., 2019).

Without external input, the cortex has a complex, ever evolving internal activity (Hipp et al., 2012; Mantini et al., 2007; Stringer et al., 2019). This global cortical state can be defined as the distribution of activity across all neurons at any given point in time, and because of the sheer number of neurons the cortical state is extremely high-dimensional (Spanne and Jorntell, 2015; Stringer et al., 2019). Since the neurons are connected to each other, the network structure will constrain the number of possible states, or at least make some states more or less likely than others (Berkes et al., 2011; Golub et al., 2018; Luczak et al., 2009). This can be described as a system which potentially has an infinite number of input-output solutions, but which have a discontinuous landscape of solutions, that is, where some solutions are much more probable than others, as in a state attractor (Ringach, 2009). That would mean that for a given tactile input pattern, for example, which is delivered randomly in relation to the current cortical state, there would be a tendency to form specific clusters of response types, or network solutions. This is, indeed, also possible to observe (Norrlid et al., 2021).

If the neocortex is sufficiently densely interconnected, an activity change in almost any individual neuron, or small group of neurons, would equal a change in the global cortical state, which, in turn, could impact the response to a given sensory input in any individual cortical neuron. Indeed, previous analyses have shown that the perturbation of the activity even of single neurons will impact the responses of other nearby neurons (London et al., 2010) and intense single neuron activation can affect behavioral decisions (Houweling and Brecht, 2008; Voigt et al., 2008).

It should also be the case that the perturbation of the activity of any neuron is theoretically possible to detect from any other single neuron regardless of its location in relation to the perturbation. The response of an individual neuron to a given input could be impacted even if the remote perturbation does not lead to any overt measurable response in it. This is because the perturbation could result in dynamic alteration of the global physiological network structure, that is, the changes in the effective network structure that would result if some neurons dynamically fall below their activation threshold, which, in turn, could impact the number of “open” network pathways that supply the neuron with inputs. Hence, an externally imposed perturbation could induce “new” states in the cortex, that is, states that does not otherwise arise, which should result in that the response states to a given input pattern deviate from the normal condition, at least temporarily, for as long as the effect of the perturbation lingers in the global cortical system. Clarifying such operational principles could give us important clues as to the properties of cortical processing. This understanding would in addition be useful for identifying limiting factors for successful communication in brain–machine interfaces. Here we explore the hypothesis that even remote temporary activity modifications could cause widespread dynamic changes in the effective cortical network structure, thereby impacting the set of network solutions to any given tactile input. We test this hypothesis by applying electrical, remote cortical perturbations while recording the intracellular responses of SI neurons to a set of given spatiotemporal tactile input patterns.

## Results

**Figure 1:**
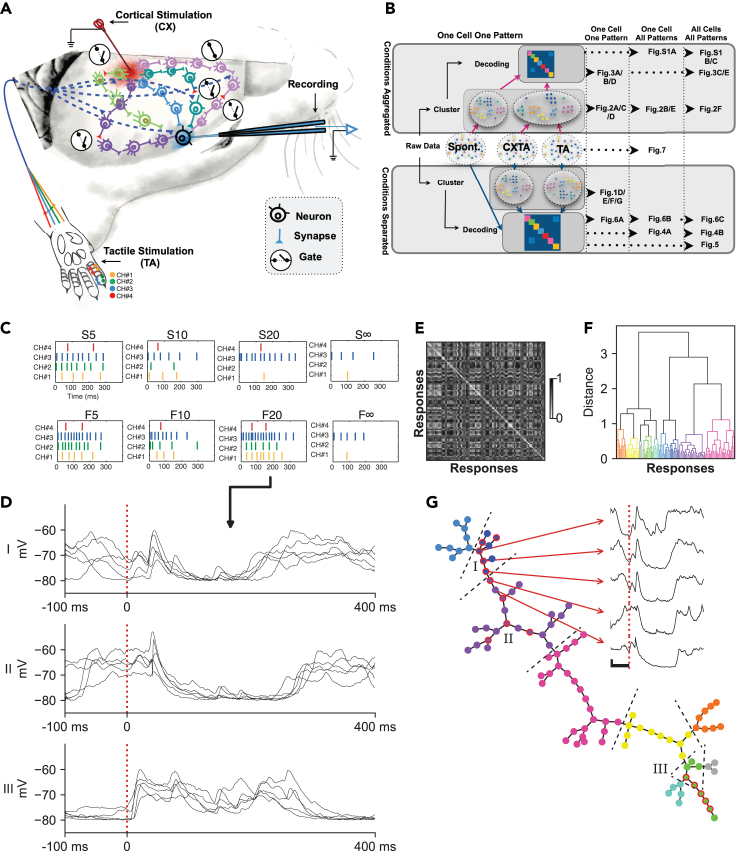
Experimental setup, analysis overview and illustration of the clustering method (A) Experimental setup illustrated in a schematic drawing. The second digit of the left forepaw was presented with eight spatiotemporal tactile afferent (TA) input patterns, delivered through an electrotactile interface across four skin sites on digit 2 (channels, indicated with different colors) (bottom). Recordings were made in the SI cortex. The TA input patterns were delivered alone or conditioned by a CX at a remote cortical site. In addition to the direct cuneo-thalamocortical input, the evoked tactile afferent activity distributes across the cortex through various neuronal network pathways, and the CX was intended to interfere with the transmission through these indirect routes (gating) before they reached the recorded neurons. (B) Overview of the analysis of the entire paper with keys to indicate which Figure shows which part of the analysis. The analysis was iterated for two different main hypotheses: (i) that the CX did not create unique clusters of responses for any given tactile input pattern and the responses evoked under the TA and CXTA conditions were therefore pooled before clustering (“Conditions Aggregated”); (ii) that the CXTA and TA conditions evoked responses that were not part of the same distribution (“Conditions Separated”). Note also that each analysis result could represent one cell and one input pattern, one cell across all input patterns, or all cells across all input patterns. (C) In the diagrams of the labeled TA input patterns, each vertical dash represents the timing of a stimulation pulse, and the color indicates through which channel it was delivered. (D) Example intracellular responses evoked by the same tactile input pattern (F20, the TA condition only), in one of the neurons. Each of the three panels show a subset of responses (N = 5) from one cluster each (I–III). Onset of the TA input pattern is at time zero, marked with a dashed vertical red line, note that the duration of this specific pattern was 320 ms (as shown in panel C). (E) Distance matrix for all responses evoked by the TA input pattern F20. Note that the matrix is symmetric, and the upper triangle of the matrix is only included for visual clarity. (F) Dendrogram based on the distance matrix in (E) visualizing the cluster formation based on the estimated optimal cutoff (see STAR Methods). Each response is colorized according to their designated cluster. (G) Proximity graph based on the dendrogram in (F). Each response (node) is connected to the closest response as defined by the calculated linkage matrix (see STAR Methods). Each response is colorized according to its designated cluster (same colors as in F). Inset, five example responses spanning one cluster boundary, with the onset of stimulation indicated by a vertical dashed red line and calibration bars indicating 5 mV and 100 ms, respectively. The raw traces illustrated in (D) are indicated as nodes with red borders (i.e., subsets of clusters I–III). Dashed lines indicate boundaries between clusters.

We recorded intracellularly from 19 putative pyramidal neurons between layers 2/3 and 5 (recording depths: 0.3–1.1 mm from the cortical surface) in the primary SI, using the *in vivo* whole cell patch-clamp technique (Figure 1A). Because two of the neurons did not have an apparent response to tactile stimulation patterns, only 17 of these recordings were included in the main analysis, whereas the two nonresponsive neurons were included in one control test (see Figure S1C below). During recordings, we delivered a set of eight spatiotemporal tactile afferent activation (TA) patterns, or input patterns, each repeated in the order of 50–100 times (see STAR Methods for details), in random order. While such tactile inputs can reach the SI neurons through direct cuneo-thalamo-cortical pathways, information about the tactile input is widely distributed in the cortex and could hence reach the recorded neuron through multiple parallel pathways (Figure 1A). To test how interference with remote cortical networks could impact the responses in the SI neurons, we combined the TA inputs with remote cortical stimulation (CX). As illustrated in Figure 1A, a localized CX perturbation may affect multiple cortical and thalamic network pathways, hence affecting their transmission state (“Gates”). If such pathways would be partly responsible for mediating the tactile information to the recorded neuron, then there would for each given tactile input pattern be multiple alternative response types that could arise in the recorded neuron depending on the combined transmission states of all pathways impacting the neuron. This transmission would, in turn, be possible to manipulate through remote cortical perturbation.

### Introduction to the cluster analysis

We used a cluster analysis to identify to what extent alternative response types/response states could be evoked by the exact same spatiotemporal tactile input pattern. The cluster analysis was performed on the responses (time–voltage curves) evoked by the TA input patterns and by the combined cortical and tactile, CXTA, input patterns, respectively. In order to establish whether clusters of responses were separable from each other, we used a decoding analysis. As shown in Figure 1B, this two-stage analysis was applied to test two different hypotheses, in turn. The first hypothesis was that CX did NOT impact the intracellular neuronal responses to a given TA input pattern, hence the responses evoked by the corresponding TA and CXTA stimulations were pooled (“Aggregated” in Figure 1B). The second hypothesis tested was that the CXTA stimulation condition created a set of responses that were separated (“Separated” in Figure 1B) from the TA responses, even though the TA input pattern component was identical between the two conditions. First, we introduce the clustering procedure by using the example of the responses evoked by one of the TA inputs patterns (Figures 1D–1G).

In order to limit the analysis to the synaptic inputs each neuron received, the recordings were made with a mild hyperpolarizing bias current to minimize spiking activity. Hence, the intracellular voltage of the single recorded neuron reflected the summed synaptic responses of all neurons being afferent to the recorded neuron. Different response states in the recorded neuron would then indicate that the states/excitability in the subnetworks controlling the activity of those afferent neurons varied differentially over time (Norrlid et al., 2021). Indeed, even though the exact same input pattern (“F20,” see Figure 1C) was repeatedly delivered, the individual raw responses could differ substantially from each other (Figure 1D). However, although the responses could vary extensively, there was also a tendency for some responses to be more similar to each other, suggesting that some preferred response states, or types, did recur (I, II, and III, respectively, in Figure 1D). The clustering analysis quantified this tendency (Figure 1E) and found that for all the responses it was a general rule that some responses, again evoked by the exact same input pattern, were more closely related to each other than they were to the rest of the responses (Figure 1F). As shown in Figure 1G, each cluster (or response type) had different numbers of “members.” “Adjacent” responses could have a somewhat gradual change in appearance, but border transitions between two separate response types were characterized by the loss or addition of distinct response features (Figure 1G, raw traces), suggestive of a dynamic gating-out or gating-in of specific subsets of the network pathways mediating the tactile input (Figure 1A).

### Impact of combining the tactile afferent activation input with cortical stimulation

**Figure 2:**
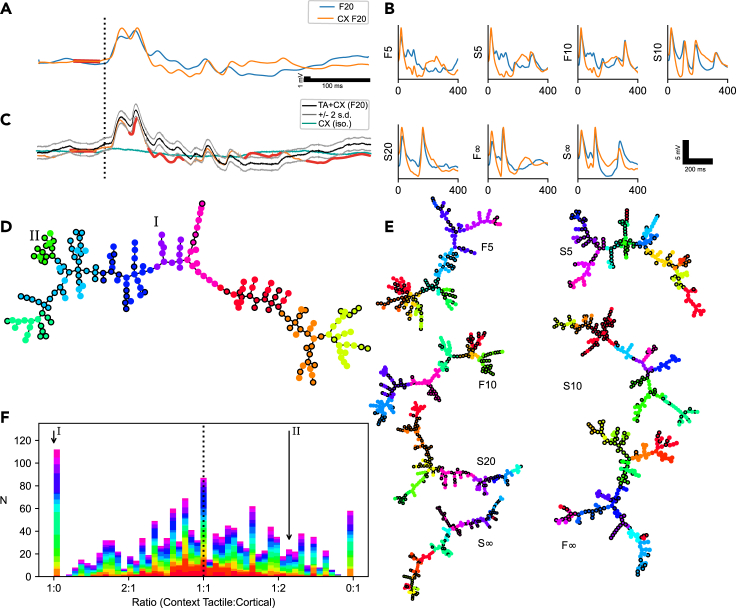
Impact of CX stimulation on TA responses and the resulting sorting into response clusters (A) Mean response to TA input pattern F20 with and without preceding cortical stimulation (CXF20 and F20, respectively). The onset of the TA input pattern is marked with a vertical dashed line. The CX stimulation occurred at the thick red horizontal line (stimulation artifacts blanked). (B) Mean responses for the other seven TA input patterns with the TA (blue) and CXTA (orange) conditions superimposed. In this case the pre-stimulus activity has been clipped. (C) Average CXTA response (orange) superimposed on the calculated algebraic sum of the TA and the CX responses (black). Thick red parts of the orange trace indicate where the CXTA response went outside the 95% confidence interval (the ±2 standard deviation range (gray traces)) of the calculated response. Blue trace indicates the CX response in isolation. In this panel only, non-smoothed responses are shown. (D) Proximity graph for the clusters identified for the aggregated F20 and CXF20 responses. The CXTA responses are indicated with black outlines. Two clusters have been marked; (I) is a cluster with only TA members; (II) is a cluster dominated by CXTA members (with a ratio of TA:CXTA responses of 1:3.6). (E) Corresponding proximity graphs for the seven other stimulation patterns of the example cell. (F) Histogram of the ratio of TA vs CXTA conditions for every single cluster from all recordings (the clusters from all 17 neurons for 8 stimulation conditions each). Each neuron is represented by a unique color. The locations of the clusters (I) and (II) from panel (D) are marked with arrows.

We next applied the remote cortical perturbation in order to characterize how it changed these response states available within the cortical network. First, when the F20 TA stimulation pattern was conditioned by a preceding CX stimulation (the CXTA condition, indicated as CF20 in Figure 2A), the average response changed. Although there was some overlap in the initial components of the two mean responses (TA versus CXTA), later components diverged more clearly (Figure 2A). Also, across the other seven TA patterns, the CXTA condition appeared to impact the average responses, again in particular the later components of the responses (Figure 2B). Notably, the CX stimulation on its own did not have any major response in the recorded neuron (Figure 2C, blue trace), which was to be expected for a remote stimulation. Moreover, as a control, we directly tested if the CXTA response deviated from the algebraic sum of the TA and the CX responses. We found that the average CXTA response fell outside the summed TA+CX response by more than 2 standard deviations for more than 5% of the response time in all cases except one (for the remaining cases, we recorded a range of 8–77% of the time spent outside the ± 2 s.d range; N = 88 cases of comparisons, based on data from the 11 cells which included the CX stimulation, across 8 stimulation patterns). Hence, these findings were compatible with that the remotely located CX stimulation (Figure 1A) primarily caused indirect interference with the subnetworks mediating the TA inputs to the recorded neurons.

### Cluster analysis for the two stimulation conditions “aggregated”

Hence, the average response evoked by each input pattern was different depending on under which condition it was evoked (TA or CXTA). However, as shown in Figure 1, these average responses were internally composed of a wide variety of response types and the main question asked in this study was to what extent the response types were impacted by the remote cortical perturbation (CXTA). Our analysis first had the working hypothesis that there was no difference between the response types evoked by specific input patterns under the TA and CXTA conditions. Therefore, for each of the eight input patterns, we pooled the responses evoked under the TA and the CXTA conditions and then applied the cluster analysis method (Figures 1E and 1F). If the response types evoked by a given tactile input pattern depended on the condition there would then be some response types that were dominated by one of the conditions, in which case this first hypothesis would be falsified. Figure 2D illustrates the clustering of the responses evoked by the F20 pattern, under both the TA and the CXTA conditions. One of the clusters (indicated by I in Figure 2D) was only populated by responses evoked under the TA condition. Although all other clusters in Figure 2D did contain both conditions, the ratio between the two conditions varied substantially between clusters, where for example the cluster II was strongly dominated by the CXTA condition. Figure 2E illustrates the same analysis but for the seven other input patterns, in which cases there were several clusters in which only responses evoked by one of the conditions were found. The illustrated cell had a total of 97 clusters (12.1 clusters in average per input pattern), of which 5 clusters contained TA responses only and 3 clusters CXTA responses only. Figure 2F summarizes the results of this cluster analysis for all stimulation patterns across all cells. From this analysis, it is clear that the majority of the clusters did contain both conditions, though with a ratio between the conditions that varied substantially, but there was a disproportionate number of “single condition clusters.” In Figure 2F, the average ratio was almost exactly 1:1, that is, 0.49 (slight bias toward ratio 1:0), with a standard deviation of 0.26. Hence, the likelihood that the ratio would be 1:0 was 0.031, which was equivalent to 44.5 clusters, and for the ratio 0:1 the likelihood was 0.027, or 38.4 clusters. However, the actual numbers were 112 and 58 clusters, respectively. Hence, these results falsified the hypothesis that the response clusters did not depend on the condition under which the response was evoked.

### Decoding analysis to verify the separability of the clusters

**Figure 3:**
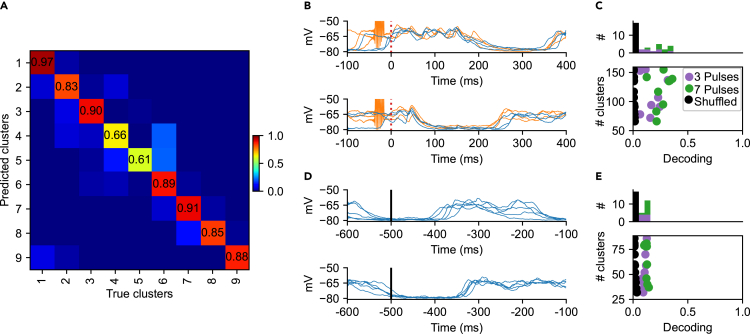
Decoding analysis to quantify the separability of the response clusters (A) Confusion matrix generated through PCA and subsequent kNN classification of clustered responses evoked by the F20 and the CXF20 input patterns (i.e., the TA and the CXTA conditions pooled) for the example neuron. The F1-score in this example was 0.85 with a chance level of 0.11 (1/9). (B) Superimposed raw traces from two example response clusters (Clusters 1 and 7) for responses evoked under the TA condition (blue traces) and under the CXTA condition (orange responses). The CX stimulation artifacts, that preceded the onset of the tactile input pattern (red dashed line), are clearly visible in the orange traces. (C) The sum of the number of clusters for each neuron (N = 17) across all eight patterns (TA and CXTA pooled), plotted against its average decoding performance (cluster separability). Neurons for which we used seven CX pulses are indicated in green, whereas neurons with three CX pulses are indicated in purple. Black dots indicate the controls, one for each neuron, where the cluster labels were shuffled. Note that the Y axis does not start at zero. The histogram on top shows the frequency distribution of the different decoding levels. (D) Examples of clusters identified in the spontaneous activity, which were recorded in the time window before the onset of the TA input pattern (−500 to −100 ms). (E) Similar display as in (D), but for clustered spontaneous activity.

In clustering approaches, there is in general a risk that the identified clusters represent a continuum rather than sets of distinctly separated data points. In order to verify that our clustering method did not suffer from this problem, we next used a PCA+kNN decoding analysis to quantify the separability of the response clusters. If the observed clusters were not separable, but instead only arbitrary boundaries drawn across noise variations in the responses, the decoding analysis would help identifying that. In contrast, the result of this decoding analysis indicated that for the example neuron and the example input pattern F20, the separability, or the decoding accuracy, of the clusters was high (Figure 3A). Note that the clustering algorithm had identified 9 clusters for this input pattern, and the axes in Figure 3A indicate which cluster was predicted (i.e., for each response, if it was a member of cluster#5 it would predict its “neighboring responses” in the principal component space to be members of cluster#5, too) and what was the actual, true neighboring clusters observed. Hence, the observed high values of the matrix elements in the diagonal of the matrix indicate a high separability of the clusters, whereas the number of misclassified responses, non-zero values outside this diagonal, was low. The average F1 score, which we used to quantify this separability, was 0.85 in Figure 3A. Figure 3B illustrates raw data from two of the response types/clusters/states in Figure 3A, where it can be seen that response states under the TA and the CXTA conditions could, indeed, in some cases be highly similar.

Figure 3C illustrates the average F1 score across all of the eight input patterns for each cell. In this case, the number of responses that each response was compared to was much, much higher than in Figure 3A, so the F1 score therefore was expected to be substantially lower than in Figure 3A, which was also the case. It can be noted that the number of clusters identified for each cell could vary substantially (60–152), but this did not appear to impact the separability (decoding) of the clusters (linear regression N = 17, Slope = 58.9 (95% confidence interval (CI) = −81.6–199.4) R2 = 3.1%, p = 0.5). Figure 3C also illustrates the shuffled control decoding for each cell, where the cluster ID# were shuffled between the responses evoked by the same input pattern before the PCA + kNN analysis was reapplied. Since the decoding accuracy collapsed following the shuffling, the identified clusters were verified to be separable by the PCA+kNN analysis. The number of pulses used in the CX condition (3 or 7, illustrated in different colors in Figure 3C) did not significantly affect how many clusters were observed (Mann–Whitney U, U-statistic = 31.5, p value = 0.35).

### Clustering analysis of the spontaneous activity

The clustering algorithm also identified response clusters in the spontaneous activity (Figure 3D). That also the spontaneous activity contained separable clusters was not surprising, since it merely indicates that the spontaneous activity does not follow fully random time evolutions, as previously reported (Berkes et al., 2011; Luczak et al., 2009). But a critical issue was whether the clusters of the spontaneous activity were broader, less specific than the clusters among the evoked responses. Indeed, the separability of the clusters of spontaneous activity was substantially lower (F1 score 0.11 ± 0.016, Figure 3E) than for the clusters formed among the evoked responses (F1 score 0.22 ± 0.09 Figure 3C). In addition, when all the clusters found for all stimulation conditions and the spontaneous activity were combined, the decoding analysis indicated a much higher separability for the evoked response types compared to the spontaneous activity clusters (Figure S1), indicating that the evoked response types were much more specific/separable than the spontaneous clusters. Figure S1C also illustrates that the two neurons without detectable levels of TA inputs were clearly singled out as nonsense information by the PCA + kNN verification method, strengthening the conclusion that the decoding analysis was an important verification of the separability of our clusters.

### Response clusters with input conditions separated were highly distinguishable

**Figure 4:**
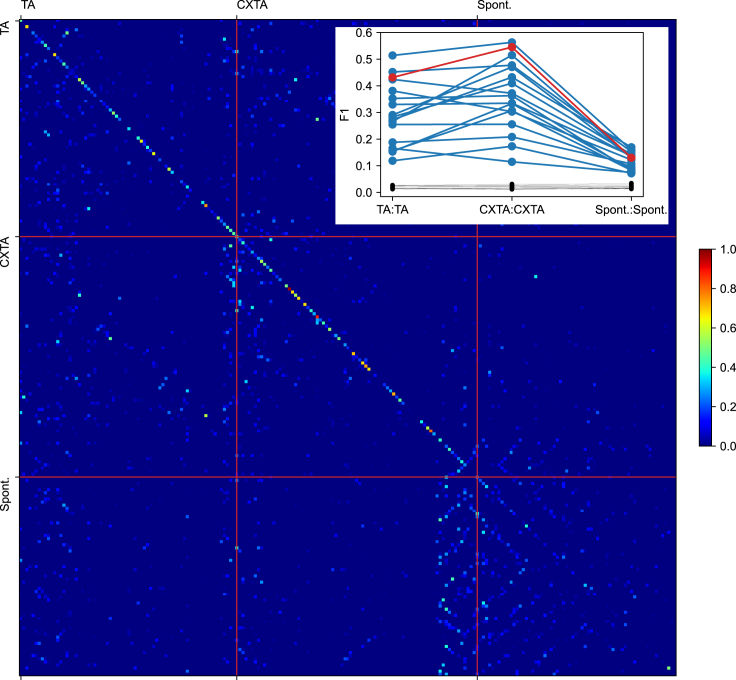
Response clusters formed based on the input condition were highly separable The separability of identified clusters when compared to all clusters from all input patterns and both conditions (separated), as well as all clusters from spontaneous activity, for the example neuron. Red lines indicate boundaries between the conditions. Inset at top right shows the F1-scores under the separate conditions for each neuron. Black lines indicate the respective chance levels. The example recording is indicated with red line in the inset.

Because the initial hypothesis tested, that is, that the set of response types evoked by each given input pattern did not depend on the condition, was falsified in Figure 2F, we next applied the combined clustering and decoding analysis for the responses that were split up depending on the condition (“Separated” in Figure 1B) (Figure 4). This giant matrix illustrates a high degree of separability of the response clusters that was obtained across all input patterns and all conditions separately, in the example neuron. The high degree of separability was remarkable given the very high number of clusters with which any given response could be confused with. The response clusters from the TA, the CXTA and the spontaneous conditions were grouped in this matrix so that the separability of these clusters could be better visualized. Within each group of clusters, the F1 score (chance level) was for TA:TA 0.43 (0.014), for CXTA:CXTA 0.55 (0.013), and for Spont:Spont 0.13 (0.015). Hence, the clusters generated by the TA or the CXTA inputs were highly separable, indicating that these condition-specific clusters were to a large degree separable from each other, whereas the clusters generated for the spontaneous activity were as a rule much less separable. Across the population of neurons (inset in Figure 4), it was a consistent pattern that the two input conditions were highly separable, whereas the spontaneous clusters were less so. Interestingly, the clusters of the CXTA condition were more distinctly separable than those of the TA condition. This could be due to that whereas under the TA condition, the tactile input patterns combined with an uncontrolled spontaneous activity, the CX stimulation may have constrained this spontaneous activity to a somewhat tighter range, hence making the CXTA response clusters more separable.

**Figure 5:**
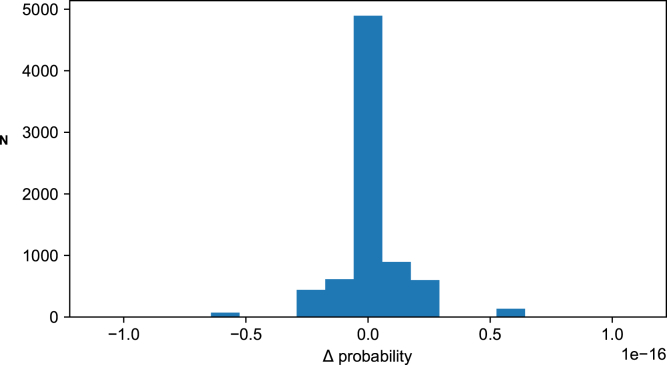
Preceding spontaneous response type did not predict evoked response type Distribution of the difference between Posterior (evoked)-Prior(spontaneous) probability of clusters. For each spontaneous cluster and each evoked cluster there was a probability given by the data, that is, the fraction of responses that belonged to each cluster. Hence, for each combination of spontaneous cluster and evoked cluster, there was a specific expected probability by which this association would occur by chance. We calculated the difference between the prior probability of observing a specific cluster, and the updated posterior probability of observing the same cluster given that we knew which spontaneous cluster that preceded it. If this difference would have deviated from zero, then this would be indicative of the spontaneous cluster being predictive of the evoked cluster. The bar chart shows that this was not the case. The Grand Mean difference between the posterior and the prior probability for all recordings, patterns, and clusters, was 1.48 × 10^−18^.

Note that the spontaneous activity was first split up depending on which TA input pattern it preceded and then clustered separately—so the confusion matrix actually compares spontaneous clusters preceding different specific TA input patterns. Consequently, confusion between the different spontaneous clusters (elements outside the central diagonal in the matrix) mostly arose with other clusters of spontaneous activity. Hence, this part of the analysis verified that when responses were similar to each other, they would also be confused and thereby collapsing the F1 score reported by our analysis. Indeed, when we compared the normal spontaneous activity with the spontaneous activity that followed the CX stimulation alone, we found that the latter was somewhat different from the former (Figure S2). We also explored if the preceding spontaneous activity cluster had a predictive influence on the response cluster, but we found no such effect (Figure 5). This result is also in line with our previous results (Norrlid et al., 2021), that neither the ECoG state nor the internal neuron state (up/down or asynchronous) had any clear predictive effect on the response states for any given tactile input pattern. This could reflect that the spontaneous internal brain states of our anesthetized preparation, while being multi-dimensional and therefore having a high number of potential solutions (Norrlid et al., 2021), contains zero prediction of the input and thereby the time evolving TA input patterns are allowed to have a gradually increasing impact on the brain state. Hence, the analysis of Figure 4 supports the hypothesis that the CXTA stimulation condition created response clusters that were at least partly separated from the response clusters evoked under the TA condition, even though the TA input pattern component was identical between the two conditions.

### Detailed decoding analysis of clustered responses with conditions separated

**Figure 6:**
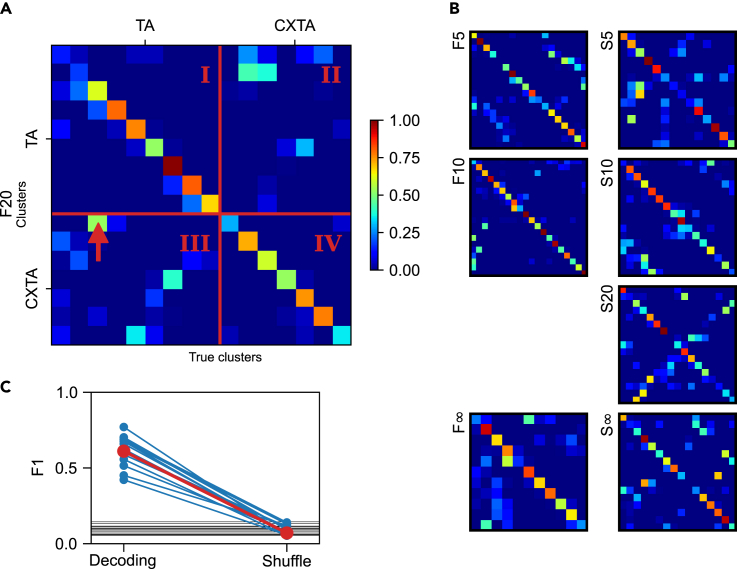
Response clusters evoked by the same input patterns but under different conditions were highly separable (A) Confusion matrix indicating the decoding of the response clusters to input pattern F20, where the clusters were formed separately for the TA and CXTA conditions (F20 and CXF20 “Separated”). The red lines indicate the boundaries between the different conditions (True:Predicted annotations are I = TA:TA, II = CXTA:TA, III = TA:CXTA, IV = CXTA:CXTA). The arrow points to an example element with confusion where the true condition (TA, according to the clustering) did not match the predicted condition (CXTA) as defined by the decoding analysis. (B) Confusion matrices similar to the one in (A) for the remaining seven tactile input patterns. (C) Average F1 scores across all neurons. The red line indicates the decoding level for all response types identified for each tactile input pattern, averaged across all eight input patterns, before and after shuffling of the response type labels, for the sample neuron shown in (A and B). The blue lines indicate the average decoding level for the other 16 neurons. The horizontal black lines indicate the mean chance level per neuron.

We next explored to what extent the response clusters evoked by each single TA input pattern under the two conditions were separable. For each given tactile input pattern, we identified the response clusters for the TA condition and the response clusters for the CXTA condition separately (Figure 1B, “Separated”), in order to compare the clusters formed under the two conditions using the decoding analysis. The diagonal of the confusion matrix of one sample input pattern in the example cell (Figure 6A) indicated a high degree of specificity of the response clusters generated separately for the two conditions. Some exceptions did occur, note for example the square indicated by the red arrow in Figure 6A, which indicated that this particular set of responses had a high risk of confusion between the two conditions, but overall, the responses were highly specific to the condition. Across the seven other stimulation patterns for this cell (Figure 6B), the risk of confusion appeared to vary substantially, with the highest risk of confusion being observed for responses evoked by the S20 pattern. On the other hand, for example, the F10 pattern seemed to generate responses that were consistently correctly classified to the respective clusters, without confusion between the conditions. Figure 6C summarizes the results of this analysis for all cells, with the results from the eight different input patterns being averaged for each cell. Hence, this analysis further supported the hypothesis that the response clusters evoked by the same input pattern, but separated on the TA and CXTA conditions, did really represent distinct cluster sets. Therefore, the remote cortical perturbation induced a sufficient change in the network state such that the input-output relationship of the recorded neurons changed, that is, the perturbation dynamically changed the network solutions to each given tactile input.

### Decoding analysis of unclustered responses

**Figure 7:**
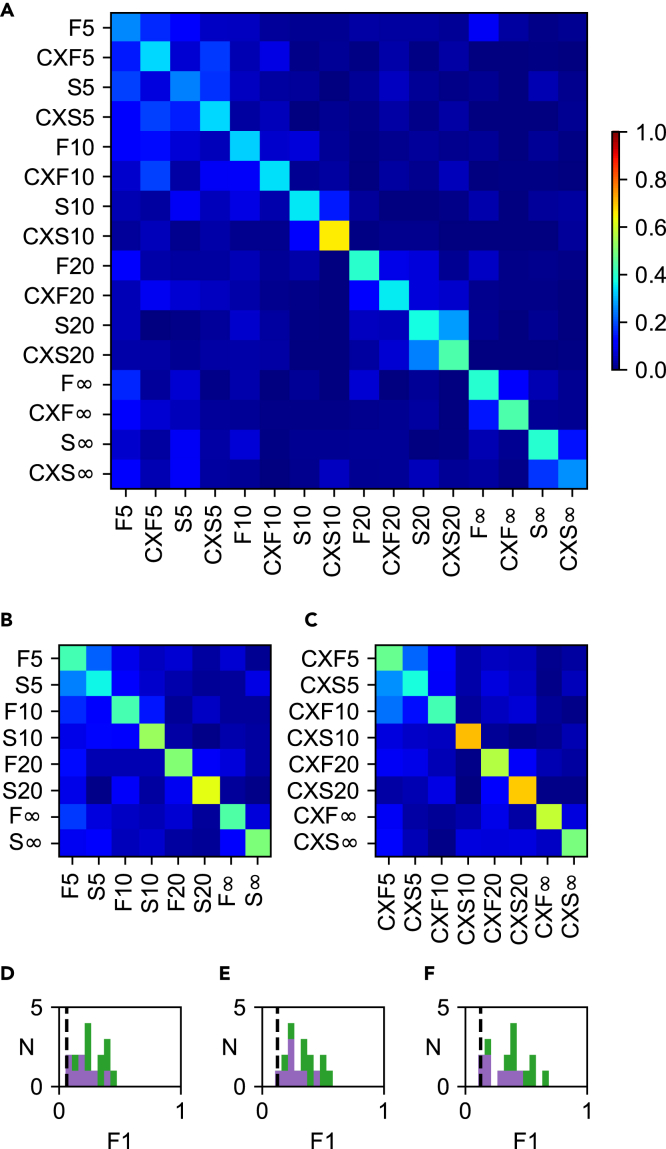
The separability of non-clustered responses was comparable across input patterns and across conditions (A) Decoding of non-clustered TA and CXTA (separated) responses for the example cell with an F1 score of 0.38 (chance level = 0.062, i.e., 1/16). (B) Decoding of non-clustered TA responses for the same cell (F1 score = 0.49; chance level = 0.125). (C) Decoding for non-clustered CXTA responses for the same cell (F1 score = 0.55; chance level = 0.125). (D–F) F1 scores for the population of recorded cells for non-clustered responses (green and purple bars indicate cells with seven and three CX pulses, respectively) for both conditions (D, F1 = 0.25 ± 0.11; shuffled control = 0.05 ± 0.004), for the TA condition (E, F1 = 0.32 ± 0.12; shuffled control = 0.12 ± 0.005), and for the CXTA condition (F, F1 = 0.36 ± 0.15; shuffled control = 0.12 ± 0.006). Chance level is indicated by the vertical dashed black line.

We also investigated if the responses evoked under the two conditions were separable without a preceding separation of the responses into clusters. Here, the question asked was if the responses evoked by the different input patterns were more dissimilar to each other than the responses evoked by the same input pattern but under different conditions (TA and CXTA). Hence, all the raw responses of the 16 stimulation conditions were compared to each other using the same type of PCA+kNN decoding analysis (Figure 7) as performed in the confusion matrices above. Figure 6A illustrates for one neuron that the un-clustered responses evoked by the different input patterns and conditions were, indeed, separable from each other, though not perfectly so; the F1 score was in this case 38% (compared to a chance level of 6.2%, 1/16). Interestingly, there were several cases where the differences between the TA responses of different input patterns (F5 and S5 for example) could be exceeded or matched by differences between the responses evoked by same tactile input pattern but under the TA and CXTA conditions, respectively (F5 and CXF5, for example). In this case, the strongest indication of that the responses evoked by the same input pattern but under different conditions were not highly separable from each other was for the S20 and the CXS20 conditions where there was a small 2-by-2 “square of confusion” formed around that part of the diagonal in the matrix. However, also in this case was the difference between the conditions greater than the similarity. Moreover, when comparing the eight input patterns for each condition separately (Figure 7B for TA; Figure 7C for CXTA) the F1 score did not rise substantially (55% and 49%, respectively, with a 12.5% chance level) compared to Figure 7A. Figures 7D–7F summarizes the result of this analysis for all neurons recorded. Overall, these results were compatible with that the differences between the responses evoked by the SAME TA input pattern under the TA and CXTA conditions could be comparable to the differences between the responses evoked by different TA input patterns under the same condition. This indicated that the TA and the CXTA responses could be as distinctly different from each other as the responses evoked by different TA input patterns.

## Discussion

We found that whereas each given spatiotemporal pattern of tactile afferent input would normally evoke one out of a relatively large set of preferred response states, the preferred sets of intracellular response states in SI neurons were systematically altered by remote, subthreshold perturbation of cortical activity. Hence, our results indicate that the cortical networks supplying each neuron recorded (Figure 1A) contain a wide range of network solutions for any given spatiotemporal pattern of sensory input and that the specific solution that apply at a given moment depends on an interplay between wide areas of the cortex, that is, the global cortical state, in combination with the actual sensor input pattern. These findings are indications of potential circuitry mechanisms underlying perception, where the internal cortical state represents “expectation,” “prediction” or, in the case that Bayesian inference mechanisms (Loeb and Fishel, 2014) are at play, the “prior.”

We have previously reported that the intracellular responses evoked in S1 neurons by the exact same spatiotemporal tactile input pattern differ from one repetition to another and that these responses tend to sort into separate preferred response types, which are unique for each neuron (Norrlid et al., 2021). Here we extended that analysis by showing that a cortical perturbation can alter the set of preferred response types/states to any given tactile input pattern. Our cluster analysis indicated that when the hypothesis was that TA and CXTA responses were not different, several clusters would be occupied only by members of one of the conditions and there were also many clusters dominated by only one of the conditions (Figure 2F). Figures 4 and 6 supported the alternative hypothesis by showing that for the response clusters evoked by the same tactile input, the presence of the remote cortical perturbation induced new response clusters that to a large extent were separable from the former. Also, without clustering, Figure 6 showed that the separability of the individual responses evoked by the same tactile input pattern under the two conditions (TA and CXTA) was comparable to the separability of the individual responses evoked by different input patterns.

Despite that the cortical perturbation could induce new network solutions, that is, input–output relationships, it did not generate distinct synaptic responses in the cortical neurons (Figure 2C) and the combination of the cortical perturbation with the tactile input pattern (CXTA) produced a response that was significantly different from the algebraic sum of the CX and TA responses. These nonlinear effects suggest that the remote cortical perturbation caused its main effect by gating pathways that mediated the tactile afferent input to the recorded SI neurons, probably a diversity of cortico-cortical pathways (Figure 1A), cortico-thalamo-cortical pathways (Halassa and Sherman, 2019) and naturally with possible involvement of subcortical structures such as the cuneate nucleus and superior colliculus.

The intracellular signal reflects the activity of the ∼10,000 neurons synaptically connected to the recorded neuron. The transmission of the tactile afferent information through each of these afferent neurons could in principle by independently controlled or gated by other network pathways. The presence of multiple response clusters/preferred response states suggests that the transmission through these afferent connections instead is partially coordinated. This is a logical consequence of that the neurons are connected to each other, directly and indirectly, since such connectivity constrains the possible response combinations across the population of cortical neurons, as also observed in the awake animal (Berkes et al., 2011; Golub et al., 2018; Luczak et al., 2009). It is important to point out that the fact that the cluster analysis identified multiple preferred response states does not imply that these response states are fixed entities or the only response states possible. The cluster analysis merely indicates that there are some central response features that some responses share, whereas at least one of these features were not shared by the members of the other clusters. Superimposed on these features were more fine grain differences between responses within clusters (see raw traces in Figures 1 and 3), which with the present dataset size and clustering method could not be further separated. The several different response states observed for each input, even as we explored input merely from distal digit 2 combined with remote CX stimulation, indicate that the cortex is potentially capable of an extremely high number of input–output solutions that probably are under dynamic control of the internal, global cortical state. The PCA/decoding analysis indicated that there was a degree of orthogonality between the clusters/response types. The orthogonality indicates that the clusters reported here were distinct from distributions expected from noise, which is in agreement with our previous analysis where we also used a set of different controls (Norrlid et al., 2021). Here we added additional controls, that is, when we shuffled the response cluster labels and the decoding was lost (Figures 3 and 6), and when two neurons without any detectable response to the tactile inputs also failed to report any significant decoding across the identified clusters (Figure S1C).

We found a discontinuous landscape of solutions also for the spontaneous activity, but these clusters of the spontaneous activity were much less separable than the response states evoked by the TA inputs (Figures 3 and 4). This can be explained by that the relatively long-lasting tactile input patterns induced more specific subsets of states in the network than the spontaneous activity, indicating that the effective network structure/cortical state interacted with and continually adapted to the time evolution of each tactile input pattern.

### Limitations of the study

It is inevitable that the anesthesia to some extent limited the range of possible spontaneous activity states. We do not think that this invalidates the main observed principle that the cortical perturbation tended to shift the state of the global network such that the range of solutions to a given sensory input pattern would alter. This notion comes from an analysis of the multiple intracellular response states induced by the same TA input patterns used here (Norrlid et al., 2021), where we found that the response states arose both during the synchronized ECoG state, the probability of which is increased by the anesthesia, and during desynchronized activity, which is more similar to the predominant state observed in awake animals (Constantinople and Bruno, 2011). Furthermore, pure neuronal connectivity considerations would seem to make the functional consequences we observe here inevitable, regardless of level of alertness. Even early estimates of the anatomical neocortical circuitry structure indicate that any neuronal signal can reach any other neuron within just a few synapses (Arbib et al., 1998)—since then, more advanced neuroanatomical techniques have revealed that the extent of cortical neuronal axon branching have been widely underestimated in the previous literature (Gerfen et al., 2018) (https://www.janelia.org/project-team/mouselight; https://mouse.braindatacenter.cn/).

Our CX does not reflect normal activation patterns within the brain and may hence push the global network into temporary states it would otherwise not enter. This is, in fact, the case for any method using any form of active interference with the cortical activity, including Brain Machine Interfaces (Niketeghad and Pouratian, 2019). But the main principle we describe that any variation in cortical activity, for example due to changes in “thoughts” or external cues from the same or from other modalities, will impact the response to a given sensory input, should anyway be valid.

### Relationship to the literature

There are several previous studies that show that the internal state of the cortex will affect the responses to sensory inputs (Arieli et al., 1996; Curto et al., 2009; Fiser et al., 2004; Hasenstaub et al., 2007). A difference with the present study is that our main aim was to show that the relatively distinct set of network solutions to a given input, or preferred response states, which the above studies did not explore, were impacted by remote cortical perturbation. The finer grain resolution that allowed the identification of these specific network solutions were due to that we had a more highly resolvable set of inputs, and in addition a highly resolved temporal response evolution across several 100 ms of continuous input (Norrlid et al., 2021). Another difference is that we did not define the cortical state by any arbitrary measure but instead let the data indicate the presence and number of response states that could arise, whereby the resolution of our state analysis could also be higher. Each neuron “sees” unique circuitry components of the global state, which can also be shown by that each neuron generates responses that are complementary to the responses of other neurons to the same input (as shown for tactile input patterns by (Oddo et al., 2017); and for visual input (Stringer et al., 2021)). Each of these circuitry components may behave somewhat differently over time, which would create the response states we observed, and which was the reason for the proposal of the concept of the “multi-structure” cortical state (Norrlid et al., 2021). We believe that these multi-structure cortical states are highly dynamic phenomena, that would risk to be overlooked following any form of response averaging, and were hence so far not well accounted for in the literature.

### Concluding remarks

Our results provide insights into how perceptual processes in the cortex can work at the neuronal circuitry level. The combination of a tactile input pattern with each neuronal response state, which would equal a component of the “prior,” “expectation,” or “prediction” currently residing in the neocortical network, will define the resulting activity distribution across all neurons of the cortical circuitry—which is a potential definition of a “percept.” Our results indicate that the neuronal response states can be made even more diversified by specific activation patterns in remote cortical areas. Hence, any internal state variation, potentially a “thought,” or any external cue, no matter how small, can potentially impact the activity distribution resulting from any given tactile input pattern, which provides for an extremely rich, high-dimensional perceptual capability. The principle of perceptual constancy, that is, a constant percept despite differences in the underlying sensory input pattern (Garrigan and Kellman, 2008) (see, also Purves et al., 2011), requires that the perceptual decision criteria are robust to a wide range of resulting states, that is, that multiple network solutions are perceived equivalently. How that process is coordinated mechanistically is a crucial issue for future studies, but it is clear from the present study that the mechanisms involve rich dynamics engaging large parts of the cortex.

## STAR★Methods

### Key resources table

**Table:** 

REAGENT or RESOURCE	SOURCE	IDENTIFIER
**Deposited data**		

Recording data	This study	https://doi.org/10.6084/m9.figshare.17032211

**Experimental models: Organisms/strains**		

Sprague-Dawley WT	Taconic	N/A

**Software and algorithms**		

Spike2	CED	ced.co.uk
MATLAB	MathWork	https://www.mathworks.com/
Python	Python	https://www.python.org/

### Resource availability

#### Lead contact

For any additional inquiry and information related to materials and resources used in this work Professor Henrik Jörntell is and will be the lead contact (Henrik.Jörntell@med.lu.se).

#### Materials availability

This study did not use any new/unique materials and/reagents.

### Experimental model and subject details

Adult Sprague-Dawley rats of male sex (N = 19, age: 12 ± 2 weeks, weight 250–380 g) were used in the acute experiments. Animals were maintained in the Lund University animal facilities under 12h light/dark condition. 2–3 rats were habituated in a cage of type 3H, before the experiments, and they were under ad libitum condition to have free access to food and water.

#### Institutional permission

The ethical approval for this study was received from the Lund/Malmö local animal ethics committee in advance (permit ID M13193-2017).

### Method details

#### Surgical procedures

In order to make acute *in vivo* recordings, adult Sprague Dawley rats were initially prepared in the same way as in a previous study (Norrlid et al., 2021), briefly according to the following procedure: 1) The animal was sedated by inhaling air mixed with isoflurane gas (3%, for ∼ 2 min); 2) To induce general anesthesia, a mixture of ketamine/xylazine (ketamine: 40 mg/kg and xylazine: 4 mg/kg, accordingly) was injected intraperitoneally; 3) an incision in the inguinal area of the hindlimb was made to insert a catheter in the femoral vein for continuous infusion of Ringer acetate and glucose mixed with anesthetic (ketamine and xylazine in a 20:1 ratio, delivered at a rate of ∼5 mg/kg/h ketamine). After the initial preparation steps, the somatosensory cortex (SI); was exposed by removing a small part of the skull on the right-hand side (∼2 × 2 mm), located at (from bregma): Ap: - 1.0 - +0.1, ML: 3.0–5.0. Also, another exposure (about the same size) was created in the skull to place the cortical stimulation electrode (CX), from bregma: approximately, Ap: −4.1, ML: 2.5. Furthermore, a surface electrocorticography (ECoG) electrode was placed in the rostral part of the second cortical exposure in order to continually monitor the level of the anesthesia.

The anesthetics used were chosen because they have previously been reported to not dramatically alter the neuronal recruitment order in spontaneous activity fluctuations and in stimulation-evoked responses as compared to the awake animal (Luczak and Bartho, 2012). As we have previously discussed extensively (Norrlid et al., 2021), anesthesia was required in order to achieve identical tactile stimulation patterns (where the electro tactile interface was the key, but would not be accepted in the awake animal) over a sufficiently long period of time. It also served to minimize brain activity noise caused by uncontrollable movements and internal thought processes unrelated to the stimuli. The level of anesthesia was assessed both by regularly verifying the absence of withdrawal reflexes to noxious pinch of the hind paw and by continuously monitoring the irregular presence of sleep spindles mixed with epochs of more desynchronized activity, a characteristic of sleep (Niedermeyer and da Silva, 2005). In order to prevent the exposed areas of the cortex from dehydrating, and to decrease the brain tissue movements, a thin layer of agarose (1%) was put on the exposed cortical areas. The animal was sacrificed by an overdose of pentobarbital at the end of the experiment.

#### Data collection: *In vivo* neuronal recordings

Intracellular recordings were made in the whole-cell current-clamp mode. The patch-clamp pipettes were pulled to impedances of 6–10 MΩ from borosilicate glass capillaries using a Sutter Instruments P-97 horizontal puller. The pipettes were back-filled with an electrolyte solution containing (in mM): potassium gluconate (135), HEPES (10), KCl (6.0), Mg-ATP (2), EGTA (10). The solution was titrated to 7.35–7.40 using 1 M KOH. The signal was amplified by an EPC-800 patch-clamp amplifier (HEKA Elektronik) in the current-clamp mode (bandwidth from DC up to 100 kHz). The data was digitized at 100 kHz using the CED 1401 mk2 hardware and recorded using the Spike2 software (Cambridge Electronic Devices, CED, Cambridge, UK).

In the next step, the patch pipette was inserted into the SI neocortex (Paxinos and Watson, 2006), first in a stepwise approach and with applied positive pressure to the electrode tip, in order to penetrate through the dura and the pia without blocking the electrode. The electrode was subsequently advanced slowly (∼ 0.28–0.3 um/sec) through the cortical tissue until it encountered a neuronal spike, evoked by electrical skin stimulation to digit 2 (single pulse stimulation using 1–4 channels (see below), intervals between stimulations 0.3 s). Once the spike signal magnitude dramatically increased, the positive pressure was switched to a brief negative pressure and a mild hyperpolarizing current (in the order of −10 pA) was applied to the electrode to facilitate the formation of a GigaOhm seal. The access to the intracellular space was then obtained by brief, episodical negative pressure applied to the electrode tip. Once the electrode had proper access to the intracellular environment and showed a stable signal, the data recording began. The data included in the analysis are from neurons with stable <−55 mV membrane potential in down states, with a peak-to-peak between up and down states of >10 mV and a spike amplitude of >25 mV before and after starting the protocol. All neurons recorded were putative pyramidal neurons rather than interneurons based on that they exhibited infrequent bursts of two or three spikes but had an absence of longer bursts or sustained periods of high firing (Luczak et al., 2009). Note that for each electrode track made in the cortex, before gaining intracellular access we also recorded the local field potentials (LFP) evoked by the electrical tactile stimulation, to identify the minimal latency time of arrival of the synaptic cortical responses from the skin stimulation. All recordings were made in layer II/III-V (at depths of 350–1100 um).

#### Design: electrical tactile and cortical stimulations

The experiments consisted of delivering tactile stimulation patterns through an electro tactile interface, consisting of bipolar pairs of percutaneous stainless steel needle electrodes (isolated except for the tip (0.2–0.5 mm) and inserted into the superficial part of the skin of the second digit of the contralateral (left) forepaw), and combining them with intracortical electrical perturbations. The skin had four such bipolar pairs of electrodes, each pair being a channel of tactile input (Figure 1A). The bipolar skin stimulation electrodes were delivering stimuli where each pulse was a 400 μA constant current pulse, with a duration of 200 μsec, which is about two times the threshold for activating tactile afferents using this type of stimulation (Bengtsson et al., 2013), and below the threshold required for activating nociceptive afferents (Ekerot et al., 1987).

The electro tactile interface was used to deliver spatiotemporal tactile afferent (TA) stimulation patterns. We used eight different TA stimulation patterns (F5, S5, F10, S10, F20, S20, F∞, S∞; Figure 1C) that were delivered in a preset randomized order (the same protocol as in Enander et al., 2019; Norrlid et al., 2021; Oddo et al., 2017; Wahlbom et al., 2019). Each TA stimulation pattern lasted for less than 350 ms, and consecutive TA patterns were separated by a randomized interval of about 1.8 s (Oddo et al., 2017).

Unlike our previous experiments, the present set of experiments included cortical perturbation, an electrical CX stimulation with a remote location relative to the recordings made in the SI cortex. The motivation for the specific choice of the location of the cortical perturbation electrode was: i) in order to demonstrate that the results would likely be in principle the same no matter which cortical area was perturbed, the idea was to perturb the cortex in an area located as remotely as possible from the recording area in S1. ii) to avoid the risk of introducing tissue damage in particular risking to rupture the major blood vessels and branches located along the midline and along the occipital pole. The point of maximal distance, with these trade-offs in mind was approximately 7 mm (medial ‘parietal’ cortex, Figure 1A). A glass-insulated tungsten electrode (exposed tip 100–150 um (Jorntell and Ekerot, 1999)) served as the CX micro-stimulation electrode, with its reference ground inserted into the neck muscle. The tip of the stimulation electrode was placed at the estimated depth of layer 5, since neurons in this layer have the most extensive cortico-cortical connectivity (Gerfen et al., 2018), as well as being prominent components of the cortico-thalamo-cortical pathways that directly distribute information across different cortical regions (Shepherd and Yamawaki, 2021). The CX stimulation consisted of constant current pulses of ≤400 μA and 0.2 ms pulse duration, consecutive pulses were separated by 3 ms, and we used either 3 or 7 pulses in different experiments. The current intensity and the number of pulses were selected so as to avoid that the CX stimulation evoked any detectable response in the recorded neurons in the SI cortex. A ‘CXTA’ stimulation consisted of the CX stimulation combined with a TA stimulation. As the CX stimulation in some experiments were found to create shock artefacts that lasted up to 15 ms after the termination of the last pulse, we set the interval between the CX stimulation and the TA stimulation so that the last pulse of cortical stimulation preceded the first TA stimulation pulse by 17 ms. There were 8 TA stimulation patterns (see above) plus 8 CXTA stimulation patterns, where the CXTA patterns consisted of the fixed CX stimulation combined with each of the 8 TA patterns.

We used a premade stimulation protocol consisting of the TA patterns and CXTA patterns, i.e., 16 patterns of stimulation, mixed in random order. Each protocol consisted of 50 repetitions of each pattern, i.e. 800 stimulus deliveries. For neuron recordings that lasted longer than the duration of a full protocol, we first made a recording of the responses to the CX stimulation in isolation (100 repetitions, at 1 s intervals), and then restarted the premade protocol for another 50 repetitions/800 stimulus deliveries. The material included 19 neurons, where two neurons were discarded from the main part of the analysis, due to a lack of a clearly detectable response to the TA inputs. Of the remaining 17 neurons, N = 6 neurons lasted for two full repetition sets (N = 100 repetitions) of the premade protocol, i.e., they hence received 1600 stimulus deliveries (800 TA inputs and 800 CXTA inputs). For the remaining neurons, we obtained recordings for 30, 31, 46, 49, 50, 50, 50, 68, 70, 90 and 94 repetitions of all TA/CXTA patterns. We obtained recordings of CX stimulation in isolation, 100 repetitions, for 11 of these neurons. Note that we have previously shown that even larger number of repetitions of the tactile afferent input patterns than we used here does not lead to detectable plastic differences in the responses they evoke in SI neurons (Wahlbom et al., 2019).

### Quantification and statistical analysis

#### Post processing: response filtering

In order to facilitate the post processing analysis of the responses (Figure 1B), we first created post-processing responses from the raw data. In order to define a stable baseline voltage, the recordings of the neuronal membrane voltages were linearly detrended in 10 second long segments. The detrending was a least-square fit linear detrending with breakpoints each 10 seconds. The recordings were subsequently low pass filtered using a simple moving average with a width of 1 ms. In the recordings, where occasional spikes appeared, the onset of these spikes were found using a spike shape template and a recursive fitting algorithm (Mogensen et al., 2019). The spikes could subsequently be removed from the recording data by subtracting the mean signal of the 50 nearest surrounding occurrences of the spike. Finally, the recordings were down sampled to 1 kHz. Overall, the software used in the below analysis was NumPy and SciPy, standard Python libraries in data science.

#### Definition of evoked responses

The time window included in this analysis started 5 ms after the onset of the TA stimulation (i.e., for both TA and CXTA stimulation patterns) and ended 400 ms later (some TA patterns lasted almost 350 ms, and responses could sometimes be detected at least 50 ms after the last stimulation pulse). The 5 ms gap relative to the TA onset corresponded to the conduction time from the skin stimulation to the earliest possible arrival of synaptic responses in the neocortex, as judged by the LFPs.

#### Definition of analyzed spontaneous activity

Analysis of spontaneous activity was based on the recorded neuron activity during the −500 - -100 ms time window relative to the onset of each TA stimulation (defined as time 0).

#### Clustering method

We used clustering analysis in order to identify the different response states that could be evoked on different repetitions of the same stimulation pattern (for both CXTA and TA patterns, ‘Aggregated’ or ‘Segregated’, see Figure 1B).

The purpose of the clustering algorithm was to detect distinct groups of response states, where the response state equaled the time-voltage curve of the evoked response. Evoked response states that were more similar to each other than they were to all other responses evoked by the same stimulation pattern were considered to belong to a cluster, i.e., the same group of responses. The purpose of the clustering algorithm was hence to identify groups where the ‘members’ of each group had to be internally similar, while also being distinct relative to other responses. Furthermore, the algorithm had to be able to automatically determine the number of clusters that could exist, since this could not be known *a priori*.

Each response was z-score normalized, i.e., the response mean over the whole time period was subtracted from each response and then the remainder of each response was divided by the standard deviation, and then a distance matrix was calculated for the whole set of responses. The distance matrix was populated by calculating the M number of Principal Components (PC) explaining 95% of the variance for N-1 responses. All responses were then fitted to the PCs using the least square method. The resulting PC coefficients were used to position the responses in the M-dimensional PC space. Within this M-dimensional space, the Euclidean distances between a reference response and the rest of responses were calculated. The distances between the reference response and each of the other responses were then put in a matrix (the distance matrix; Figure 1E), and the procedure was repeated until each response had been the reference. Finally, the distances were normalized.

From the Euclidean distance calculations above, a linkage matrix (Figure 1F) was calculated using the hierarchical clustering approach of Ward's minimum variance method (Ward, 1963). This method sequentially finds the two closest neighboring responses with respect to their Euclidean distance from each other, and their Euclidean distance is indicated in a dendrogram such as in Figure 1F (this ‘cophenetic distance’ is indicated as the distance along the y axis). Then it finds the pair of the second closest neighboring responses and so on until there are no responses left. However, a response may also be closer to a center point between a pair of responses than it is to other responses, in which case the cophenetic distance to that center point is assigned to the response. Sometimes, the shortest distance that can be found is between two such center points. All these distances are used to create the hierarchical structure of responses, where the distances between all such pairs can be compared (the distance along the y axis of Figure 1F).

While following the above principles, there were some special situations that could arise. A cluster was assumed to be valid only if it had five or more members. All non-valid clusters were put in the same “undefined” cluster. In addition, for one of the cells the situation arose that one of the clusters consisted of only one member. In this case this response and cluster was excluded from all subsequent analysis and displays.

The next issue is to identify the cophenetic distance at which the identified clusters are objectively the most separable. The y axis in Figure 1F (the full range of which corresponds to the largest total cophenetic distance) was then divided into 100 steps. For each level of cophenetic distance, we calculated the number of clusters found for the actual data. Data shuffling was achieved by shuffling the position of each response along the X axis of the dendrogram (Figure 1F) (The shuffling was performed using the NumPy shuffle method), and therefore disrupting the relationship between cluster and response.

We next calculated the separability of the clusters by using a decoding analysis (described in detail below). The decoding analysis yielded, for each clustering result, a measure of the separability of the clusters as the F1-score. With the F1-score, we could apply Gap statistics to identify the cophenetic distance providing the largest cluster separation relatively to the shuffled data. For each of the 100 cophenetic distances considered, we obtained a F1 score for the non-shuffled data and another F1 score for the shuffled data. Since the specific value of the chosen cophenetic distance defines the number of clusters to be considered, we could identify continuous stretches of cophenetic distances where the number of clusters remained unchanged. Over each of these continuous stretches we calculated a mean F1-score, and subtracted the mean F1-score of the shuffled data from the actual mean F1-score, which yielded the Gap statistic (Tibshirani et al., 2001). The middle value of cophenetic distance of the stretch with the maximum Gap statistic was used to define the cophenetic distance at which the clustering was objectively the most distinct. The clustering obtained at this value was then used for the remainder of the analysis and displays. The identified clusters using this approach are indicated by color codes in Figure 1F for an example cell and example TA stimulation pattern.

#### Calculation of the predictability of evoked clusters from spontaneous clusters

In order to calculate whether a specific cluster of the preceding spontaneous activity could be used to predict the evoked response cluster, we applied Bayes theorem:

ΔP = P(A)-P(A|B)

where A was the specific response type/cluster of the evoked response and B was the cluster of the spontaneous activity just preceding the onset of stimulation for the evoked response. For the clustering of the evoked and the spontaneous activity, see above, clusters were based on 300 ms time windows of continuous recording data.

#### Visualization of hierarchical proximity

Linkage matrices are commonly visualized as dendrograms (Figure 1F). They display hierarchical data in a 2D-manner, and the relationship between clusters can be easily seen. However, they fail to display the detailed relationships between the constituent responses of the clusters. For example, the gradual but distinct transformation of one cluster into another proximal cluster (inset traces in Figure 1G) cannot be seen using a dendrogram. Therefore, we also used an additional visualization technique.

The linkage matrix was transformed into a topological ‘Proximity’ graph by iterating through each defined bifurcation of the dendrogram (Figure 1F). Each bifurcation was considered to describe an edge and the branches describing the nodes connected by this edge. If a branch did not terminate in a specific response but instead into a center point between responses/clusters, then the edge was connected to the response within that cluster that had the least distance to the reference response on the other side of the bifurcation. The weight of the edge was set to be equal to the distance – 1, since they are conceptual inversions of each other meaning that a long distance is the same as having a low weight edge connecting them. The layout of the resulting proximity graph (Figure 1G) was finally optimized using the Kamada-Kawai algorithm (Kamada and Kawai, 1989).

#### Evaluation of cluster separability using decoding analysis

A general decoding algorithm to determine the specificity of the response clusters was used throughout this article. It is the same decoding algorithm used by our lab in several previous publications (Enander and Jorntell, 2019; Enander et al., 2019; Wahlbom et al., 2019, 2021), including intracellular recording data with multiple response states to each tactile input pattern (Norrlid et al., 2021).

The responses and their respective label were split into a training- and a test set stratified by cluster label frequency. The training set was used to train a PCA model explaining 95% of the variance, and the coefficients obtained by fitting the responses with the least-square-method to the principal components were calculated for both the training set and the test set. Finally, a kNN-classification was performed with the training set coefficients as training data, and the test set coefficients as test data. This decoding algorithm was performed 30 times, each time with a new split of train and test data. The F1-score was calculated from the average classification result across the 30 repetitions.

The decoding analysis was also repeated with the randomly shuffled cluster labels, reported as the “shuffled” context. This was done to establish the actual chance level of the data, as opposed to the theoretical chance decoding level (which equals 1 divided by the number of clusters). Finally, the shuffling was repeated three times to obtain the mean F1-score for the shuffled context.

## Data Availability

• The original contributions and raw data belong to this study are deposited in figshare.com and are publicly available; https://doi.org/10.6084/m9.figshare.17032211. • The supporting data is deposited at figshare.com and are publicly available; https://doi.org/10.6084/m9.figshare.17032211. • Further information about the analyzed data discussed in this paper will be provided by the lead contact upon demand.
