# Supplementary material for: Remote cortical perturbation dynamically changes the network solutions to given tactile inputs in neocortical neurons
Source: iScience. 2021 Dec 2;25(1):103557. doi: 10.1016/j.isci.2021.103557 (PMC8689199; doi:10.1016/j.isci.2021.103557)

iScience, Volume *25*

Supplemental information

Remote cortical perturbation dynamically changes the network solutions to given tactile inputs in neocortical neurons

Leila Etemadi, Jonas M.D. Enander, and Henrik Jörntell

Figure S1. Decoding analysis for the 'aggregated' clusters identified across all TA patterns, related to Figure 3.

Using clustering analysis for the data set with conditions aggregated, i.e., when the cluster analysis was based on pooled data from the two conditions (but still for each TA pattern and each cell separately) we explored if these clusters could be segregated from each other using the decoding analysis. We also included the clusters identified for the spontaneous activity in the same neuron. As shown in the resulting giant matrix (A), it did indeed turn out that the responses evoked by the different TA input patterns formed clusters that were separable from each other. However, there were a few exceptions where some response types (clusters) were not well separated from the other clusters (i.e., dark blue squares in the diagonal of the matrix). Red lines indicate boundaries between evoked and spontaneous response clusters. The F1 scores for the evoked responses were 0.49 (chance level=0.010; TA & CXTA compared to TA & CXTA) and for the spontaneous activity 0.12 (chance level=0.013; Spont. compared to Spont.), respectively. (B) Across the population of cells, the F1 score for the elements of the matrix belonging to the evoked responses was much higher than the F1 score for the spontaneous clusters (the illustrated cell in A is shown in red and chance levels are shown in black). (C) is similar to B but instead reports how many clusters (as a fraction of all clusters of the same stimulation pattern) that had an F1 score above a threshold detection level. The threshold was defined as the weighted mean F1 score plus two times the weighted standard deviation calculated from the response clusters of the spontaneous activity for each recording. In this plot, we also included the 2/19 cells that did not have a clearly defined response to the TA inputs (green). Unlike all other neurons, these 2 neurons fell down to a value close to zero in this analysis, which illustrates that neurons without meaningful clusters to TA inputs was clearly singled out by the PCA+kNN verification method. Hence, this control indicated that the PCA+kNN could be used to indicate that the identified clusters were indeed separable in the 17/19 neurons included in the analysis.

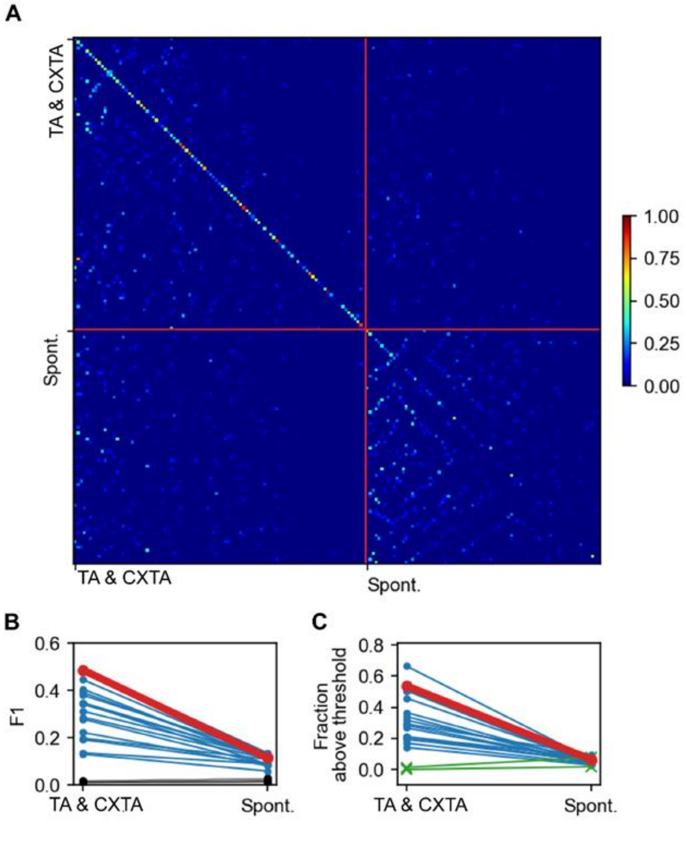

Figure S2. Spontaneous clusters were mildly impacted by the CX perturbation, related to Figure 4.

Confusion matrix of the spontaneous clusters obtained from the activity preceding the TA input patterns (N=8) and from the activity immediately following the CX perturbation (CX (iso.)). Note that the unperturbed spontaneous activity had several clusters that were moderately separable, but also that many of the spontaneous clusters were similar to each other as indicated by the low separability in the majority of cases (dark blue pixels along the middle part of the diagonal). As for the spontaneous clusters from the CX (iso.) condition, the first three of these clusters were inseparable from the unperturbed spontaneous activity (first three dark blue pixels in the diagonal below the crossing of the red lines). However, several of the subsequent, and therefore smaller, cluster groups were more distinctly different to the clusters present in the unperturbed spontaneous activity. Decoding accuracy spont:spont = 13%; Decoding accuracy CX:CX = 38%. Across all cells, the Grand Mean Accuracy for spontaneous clusters was 12.9%+/-1.8% (mean+/-s.d.) and for CX spontaneous clusters it was 27.6%+/-16.7%.

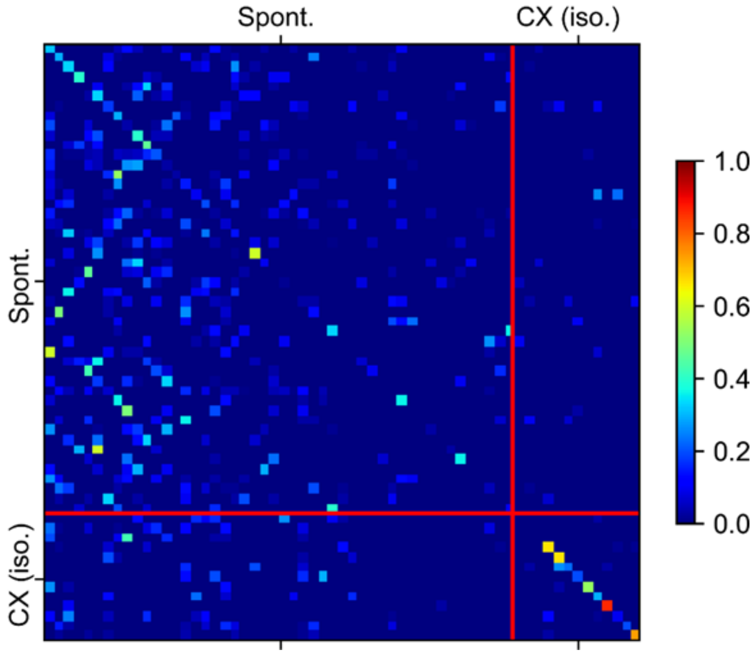

Supplement: Document S1. Figures S1 and S2 [file mmc1.pdf]
